# The Care of the Feeble-Minded

**Published:** 1893-09-02

**Authors:** 


### THE STORY OF THE INSANE FROM YEAR TO YEAR.

### THE CARE OF THE FEEBLE-MINDED.

#### I.—In Childhood.

All who have had any experience in work among the poor in large towns know what it is to find their efforts baffled by a certain deficiency, mental, moral, or physical (often all three), on the part of those with whom they have to do. The hopeless idiot is now rarely seen. His lot, provided with healthy occupation and varied amusements in asylums especially adapted for his needs, is happy enough. But there remain innumerable cases where the deficiency is not sufficiently marked to justify the term idiocy, but where from one cause or another there is an inability to profit from an ordinary education or undertake the duties of an ordinary situation in life. In every large school there is a proportion of children who, though perfectly able to take care of themselves, and even under certain conditions be useful, are yet unable to master the simplest lesson. Such children linger on in the first or second standard, incapable of attention, and at best passively enduring the long hours of inaction, until, at the age of fourteen, to the relief of the teacher, they leave school with minds as blank and fingers as helpless as at the beginning.

An excellent piece of work has lately been carried through by the Charity Organization Society in investigating the extent to which this feeble-mindedness prevails among the children of London, and in preparing a * report with recommendations grounded on the information thus obtained. The number of children examined was 50,027, and of these over 9,000 cases presented either defects in development, "nerve signs" (betraying brain disturbance), a low state of nutrition, or a marked dulness in apprehension. It is a curious fact that the symptom of low nutrition occurs in by far the greatest proportion in the schools situated in neighbourhoods where the parents are for the most part well to do, and it is argued that "not poverty, but physical defects of some kind are as a rule the chief cause of difficulty in the nurture and training of children."

All these 9,000 children (the average for the whole school population of London is estimated at 1·3 per cent.) were in various degrees unsuited for the education provided by the Board and other schools for the healthy. In other words they were practically receiving no education at all, while standing in peculiar need of the most careful training of their enfeebled powers. A minute analysis is given of the defects noticed in the course of the investigation, and a very interesting account of the method pursued by Dr. Warner in detecting them. The examination revealed incidentally "what a large amount of ophthalmic work is needed among children"; squint cases, ophthalmia, chorea, rickets, defective palate, &c., are specified as often overlooked in schools, and appear as frequent symptoms or causes of defective mental development.

The recommendations of the Charity Organization Society fall under three main heads. (1) That auxiliary day classes should be established in each large centre for "abnormal" children, conducted on the Fröbel system "modified according to the experience gained in the education of imbecile children." Such classes are in actual operation in Germany, Norway, and other countries, and one has been lately started at Leicester. The results are found fully to justify the extra expense incurred. (2) That all cases in schools requiring medical attention should be detected by means of a yearly medical inspection and receive prompt relief. The desirability of co-operation with the parents in treating ailments such as those mentioned above, is strongly emphasized, but undoubtedly presents peculiar difficulties. (3) As the workhouses cannot adequately deal with children presenting peculiarities or deficiencies, the establishment of small school homes by voluntary effort is suggested, to receive those who are entirely friendless.

All this involves special training in a suitable training college for the teachers in both the day and home schools, and a more thorough and scientific system of teaching the feeble-minded than has yet been formulated. This, and an infinite amount of adjustment to the existing Poor Law and School Board regulations, with a considerable increase of expenditure, and a large body of volunteer workers endowed with almost superhuman patience and tact. Yet it is safe to prophesy that if anything like a satisfactory future can be rendered probable for this unhappy class of little sufferers, the money and labour will be forthcoming, and at no distant time.

* "The Feeble-minded." (Swan Sonnenschein and Co.)

